# Addressing Passive Smoking in Children

**DOI:** 10.1371/journal.pone.0093220

**Published:** 2014-05-08

**Authors:** Sasha G. Hutchinson, Jennifer S. Kuijlaars, Ilse Mesters, Jean W. M. Muris, Constant P. van Schayck, Edward Dompeling, Frans J. M. Feron

**Affiliations:** 1 Department of Paediatric Pulmonology, CAPHRI School for Public Health and Primary Care, Maastricht University Medical Centre, Maastricht, the Netherlands; 2 Department of Epidemiology, CAPHRI School for Public Health and Primary Care, Maastricht University Medical Centre, Maastricht, the Netherlands; 3 Department of Family Medicine, CAPHRI School for Public Health and Primary Care, Maastricht University Medical Centre, Maastricht, the Netherlands; 4 Department of Social Medicine, CAPHRI School for Public Health and Primary Care, Maastricht University Medical Centre, Maastricht, the Netherlands; Old Dominion University, United States of America

## Abstract

**Background:**

A significant number of parents are unaware or unconvinced of the health consequences of passive smoking (PS) in children. Physicians could increase parental awareness by giving personal advice.

**Aim:**

To evaluate the current practices of three Dutch health professions (paediatricians, youth health care physicians, and family physicians) regarding parental counselling for passive smoking (PS) in children.

**Methods:**

All physicians (n = 720) representing the three health professions in Limburg, the Netherlands, received an invitation to complete a self-administered electronic questionnaire including questions on their: sex, work experience, personal smoking habits, counselling practices and education regarding PS in children.

**Results:**

The response rate was 34%. One tenth (11%) of the responding physicians always addressed PS in children, 32% often, 54% occasionally and 4% reported to never attend to it. The three health professions appeared comparable regarding their frequency of parental counselling for PS in children. Addressing PS was more likely when children had respiratory problems. Lack of time was the most frequently mentioned barrier, being very and somewhat applicable for respectively 14% and 43% of the physicians. One fourth of the responders had received postgraduate education about PS. Additionally, 49% of the responders who did not have any education about PS were interested in receiving it.

**Conclusions:**

Physicians working in the paediatric field in Limburg, the Netherlands, could more frequently address PS in children with parents. Lack of time appeared to be the most mentioned barrier and physicians were more likely to counsel parents for PS in children with respiratory complaints/diseases. Finally, a need for more education on parental counselling for PS was expressed.

## Introduction

Passive smoke (PS) exposure in children contributes significantly to morbidity and mortality [1]. The majority of disability-adjusted life-years because of PS exposure worldwide are due to lower respiratory infections in children younger than five years of age [2]. Other health effects of PS exposure in children include increased risk of higher respiratory infections, wheezing, and asthma [3], [4], [5]. Although PS exposure in Dutch children decreased since 1996, data from 2009 showed that 19% of all children until four years of age are exposed to PS at home, especially in social and economic deprived families [6]. A study involving 25 European countries by Boldo et al reported rates of PS exposure in children aged below 14 years ranging from 19% in Sweden up to 48% in Austria. In this study, Netherlands shared the sixth place together with Italy and the Czech Republic showing a prevalence of 31% [7]. Furthermore, awareness that cigarette smoke endangers the health of non-smokers appeared lowest in Dutch smokers compared to smokers from other countries [8].

The significant number of parents that seem unaware or unconvinced of the health consequences of PS exposure in children [9], provide an opportunity for health care workers to offer parental counselling to prevent PS exposure in children. However, competing priorities [10], lack of time [11], [12], anticipated negative parental response [11], [12], [13], PS exposure not considered to be part of the child health professionals' policy [14], [15] and lack of skills or confidence [10], [11], [12], [13] have all been described as barriers for physicians to discuss PS exposure in children with parents.

For a randomized controlled trial (the PREPASE study [16]), testing the effectiveness of a program to stop PS exposure in children 0–13 years of age with high risk of asthma, we asked all physicians working with children in South-Limburg (a province in the south of the Netherlands) to recruit participants. Recruitment through physicians has shown to be feasible in other studies involving children in South-Limburg [17], [18]. However, the number of recruited participants by physicians for our study was surprisingly low. This made us question to what extent physicians addressed PS exposure in children during their consultations. Therefore, we asked paediatricians, youth health care physicians (YHCPs) and family physicians (FPs) in Limburg to what extent they discuss PS exposure in children with parents. Furthermore, because of the differences between the three health professions (in terms of types of children seen during consultation, available time for consultation and postgraduate education on PS exposure in children), we also evaluated potential differences between them with respect to attitudes, facilitators, and barriers regarding counselling for PS exposure.

To illustrate these physicians' everyday practice, we will briefly introduce our Dutch healthcare system. The YHCPs and FPs generally work in the field of primary health care, while the paediatricians are typically employed in the secondary echelon of health care. The youth health care (YHC) consists of preventive health care offered to all children 0–19 years of age living in the Netherlands. YHC is provided by the YHCPs and nurses. Their role is to monitor physical, psychological, social and cognitive development of children, and to provide health information to parents and their children [19]. During 20 minutes consultations, the main focus is on early detection of potential health and developmental problems in children. According to the national guideline for asthma prevention, all YHC professionals are required to ask parents about their smoking behaviour and PS exposure in children during the consultations, when the child is 2 weeks, 4 weeks, 8 weeks, 11 months, 18 months, 5 years, 10 years, and 13 years of age [20]. In case of PS exposure, the YHC professionals are expected to give tailored health education and advice on PS exposure in children using a five-step procedure [21] developed by the Dutch expert centrum for tobacco prevention (STIVORO) [22]. FPs have 10 minutes reserved per consultation. They see children in all age groups for a variety of reasons. Based on their guidelines, FPs are also required to inquire after PS exposure in children when a child presents for consultation with respiratory complaints, and advice parents to stop smoking in the presence of their children when necessary. Both YHCPs and FPs may refer children to other type of primary or secondary health care. In the Netherlands, consultations with a paediatrician only take place after referral by FPs. The first consultation can take up to 30 minutes [23]. Although a specific guideline for PS exposure in children is not available, all paediatricians are expected to inquire and advice parents regarding PS exposure in children, especially in case of respiratory complaints.

## Methods

### Study design

We conducted a cross-sectional study using an electronic questionnaire (LimeSurvey version 1.91+ (GNU General Public License, Boston, MA, USA)) that was administered through e-mail. All paediatricians (n = 96), YHCPs (n = 82), and FPs (n = 542) of Limburg were approached. Their email addresses were collected through secretaries and associations of the three specialties. Data were collected in October and November 2011.

### Questionnaire development

Content validity: the questionnaire was based on prior studies on this subject [10], [11], [15], [24], [25] and the authors' knowledge about and personal experiences with the topic of PS in children. The definite version was based on expert agreement of all the authors who are specialists in the fields of paediatrics, family medicine, youth health care, epidemiology, and health promotion. An English version of the main questions of the survey is provided as a supplement to this manuscript. The questionnaire was proof-checked by the authors, and piloted among three independent medical doctors (face validity).

### Questionnaire content

The questionnaire consisted of 19 items (see Questionnaire S1). Physicians reporting current/past smoking received six extra items about their smoking behaviour. It took about ten minutes to complete the questionnaire. All respondents were questioned about their sex, specialisation, work experience, personal smoking habits, attitudes, knowledge, perceived skills, facilitators and barriers with regard to addressing PS exposure in children. Physicians, who reported to address PS exposure in children, were also enquired about questions asked during the consultation for PS exposure in children. Also, if they explained the health risks of PS exposure in children to parents and advised them to stop smoking in the presence of their children. Furthermore, they were asked if they had ever received postgraduate education about PS exposure in children and whether they needed (more) training on this topic. Facilitators were answered on a four-point Likert scale: very much likely (1), very likely (2), not very likely (3), and not likely (4). Applicability of each possible barrier included in our questionnaire was answered on a five-point Likert scale: very much applicable (1), very applicable (2), neutral (3), not very applicable (4), and not applicable (5). Response options for the remaining questions were categorical.

### Questionnaire distribution

An announcement with information about the study was e-mailed, followed by the questionnaire. Non-respondents received a reminder after one week. Three reminder e-mails were sent to increase response rates. Each time we emphasized that processing of the questionnaire would be kept anonymous.

### Data analysis

Data were analysed using SPSS 18. We used Chi-squared (*χ* ^2^) tests to compare the overall frequencies between the specialties for the group characteristics. Analysis of variance (ANOVA) has been found appropriate for Likert scale analysis [26]. Therefore ANOVA test was used to compare the practices of the three health professions with regard to parental counselling for PS in children. A probability (*p*) value <0.05 was considered significant. In case of *p*<0.05, pairwise comparisons were made to pinpoint the differences between the specialties. Bonferroni correction for multiple testing was applied. Missing data were addressed using pairwise exclusion. Possible confounders (the physicians' sex, years in practice, personal smoking habits, PS exposure experience during childhood, and education regarding PS exposure in children) were included as covariates in the ANOVA test analyses for the group differences concerning counselling for PS exposure in children. In case of significant contribution, they were added in the final analyses. Logistic regression analyses were performed to evaluate the association between each facilitator and barrier, and the frequency of addressing PS exposure in children. The outcome categories for “Do you address PS exposure in children”, were re-categorised into a binary outcome (always/often into “yes” and occasionally/no into “no”). The answering categories for the facilitators were recoded into two groups: very much likely and very likely into “very (much) likely”, not very likely and not likely into “not (very) likely”. The answering categories for the barriers were recoded into three groups: very much applicable and very applicable into “very (much) applicable”, “neutral”, and not very applicable and not applicable into “not (very) applicable”. The results are presented as unadjusted and adjusted odds ratios (ORs) with their corresponding 95% confidence intervals (95% CIs). The adjusted model included the next possible confounding factors: sex (male/female), profession (paediatricians, YHCPs, FPs), and PS exposure education, and current smoker (yes/no).

Furthermore, the number of facilitators and barriers reported by physicians were assessed. For these analyses, the answering categories for the facilitators were recoded into two groups: very much likely and very likely into “yes”, not very likely and not likely into “no”. The barriers were recoded into two answer categories: very much applicable and very applicable into “yes”, and neutral, not very applicable and not applicable into “no”. Additionally, the association between the amount of facilitators and barriers respectively, and addressing PS exposure in children were assessed using logistic regression analyses.

For a non-response analysis, a random sample of 30% of all non-responders received a hardcopy of an abbreviated version of our questionnaire asking them their profession, whether they discussed PS exposure in children with parents and reasons for not participating.

### Ethics

The study is part of the PREPASE study which was approved by the Medical Ethics Committee Maastricht University Medical Centre. However, this current part of the overall study was not specifically approved as it does not fall under the Medical Research Involving Humans Subjects Act, since participants were not subjected to procedures or required to follow rules of behaviour. Therefore, ethics approval for this current study was deemed unnecessary. The data were collected anonymously.

## Results

### Response and group characteristics

Table 1 shows the characteristics of the different health professions. The overall response rate was 34%: paediatricians 45%, YHCPs 73%, and FPs 26%. 103 physicians replied to the first invitation, and additionally 74, 37, 27 after the first, second, and third reminder respectively.

**Table 1:** Characteristics.

	Paediatricians (n = 43)	Youth Health Care Physicians (n = 60)	Family Physicians (n = 138)
**Sex**			
Female*	61% (26)	87% (52)	35% (48)
**Years in practice**			
Less than 3 years*	21% (9)	9% (5)	3% (4)
3 to 10 years	33% (14)	25% (15)	25% (35)
More than 11 years*	47% (20)	67% (40)	72% (99)
**Smoking**			
Current	5% (2)	3% (2)	6% (8)
Ex-smoker*	14% (6)	25% (15)	38% (52)
Unknown	2% (1)	0% (0)	4% (6)
**PS exposure as child**			
Yes	40% (17)	57% (34)	50% (69)
Unknown	2% (1)	0% (0)	4% (6)
**PS exposure Education**			
Yes*	21% (9)	57% (34)	9% (12)
Unknown	2% (1)	0 (0%)	4% (6)

Missing values: N = 4 did not provide their specialty. Other missing values are presented as unknown in the table. PS  =  passive smoke.

* = p<0.05.

### Discussing PS exposure in children

**Figure 1:**
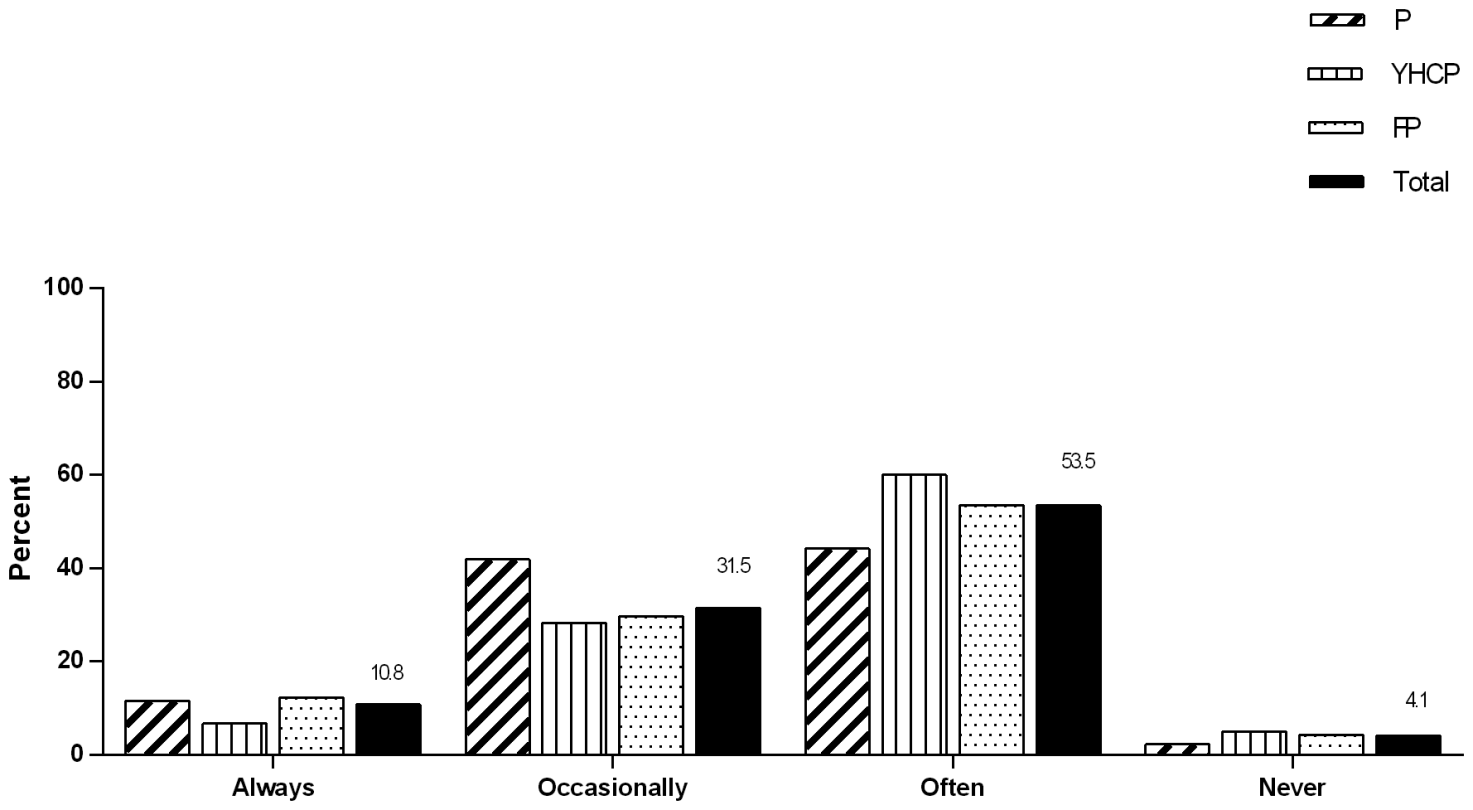
Frequency of addressing passive smoke exposure in children. The percentages of all the physicians (P  =  Paediatricians; YHCPs  =  youth health care physicians; FPs  =  family physicians) per frequency category are noted in the last columns. There were no significant differences between the three health professions (F(2,1) = 1.59, p = 0.206).

Only 11% of responders addressed PS exposure in children always (see Figure 1). Fifty four per cent reported to attend to PS exposure occasionally. There were no significant differences between the health professions (*p* = 0.206) with regard to the frequency of addressing PS exposure in children. The confounders did not have influence on these results. Only 4% of all the physicians reported to find it difficult to address PS exposure in children. Most FPs (83%) and YHCPs (77%) reported that it was their responsibility to address PS exposure in children, in contrast to the paediatricians (21%). Overall, 5% of all the physicians thought it was the responsibility of the paediatricians, 34% of the YHCPs and 60% of the FPs.

### Topics discussed when addressing PS in children

**Figure 2:**
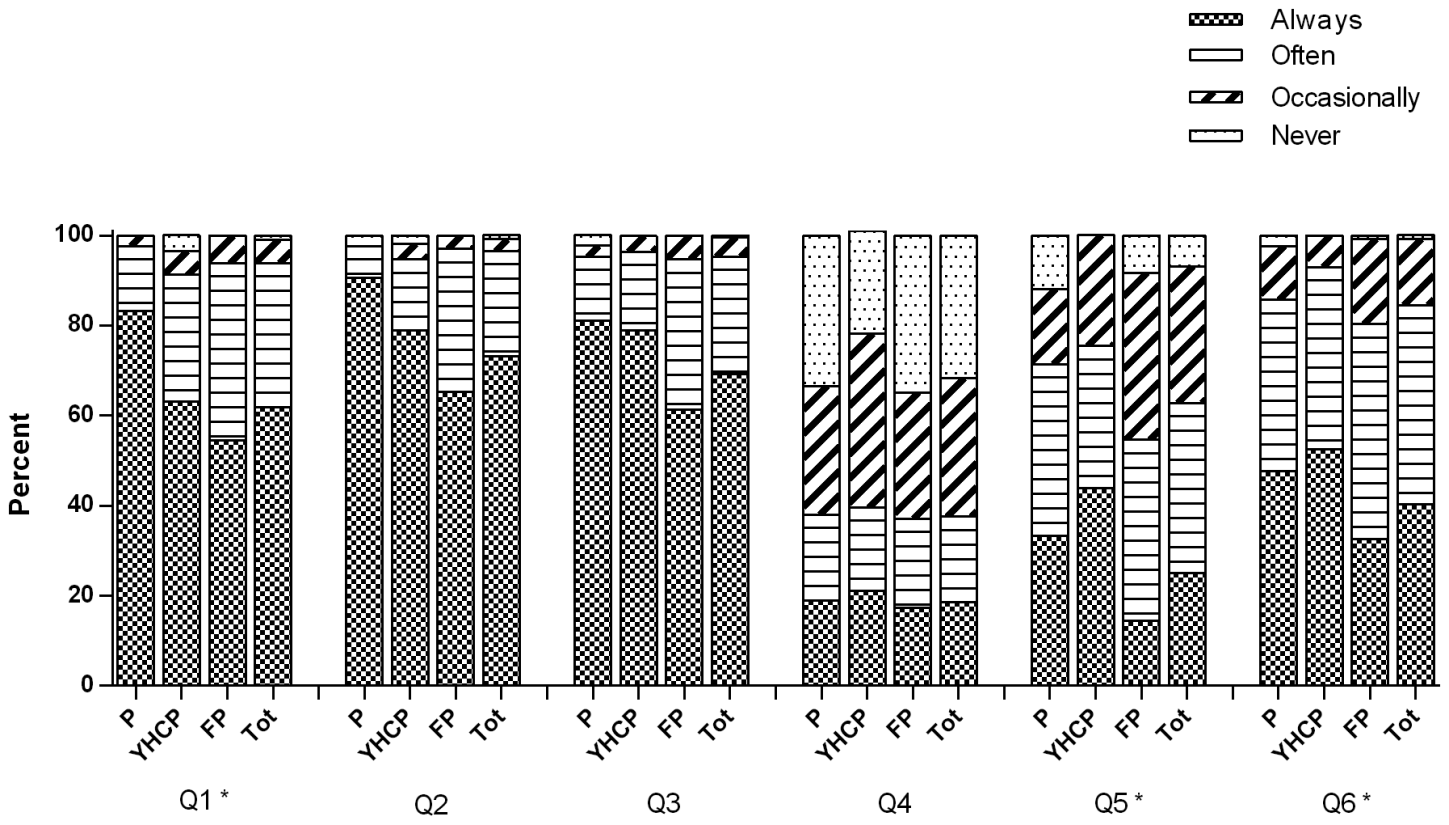
Questions asked during parental counseling for passive smoke exposure in children. Q1) Does any member of your family smoke? Q2) Does anyone smoke inside the house? Q3) Does anyone smoke in the presence of your child? Q4) Does anyone smoke inside your family car? Q5) Did you take any efforts to prevent passive smoke exposure to your child? Q6) Are parent(s)/caregiver(s) aware of the health consequences of passive smoke exposure to their child? (P  =  paediatricians; YHCPs  =  youth health care physicians; FPs  =  family physicians; Tot  =  total). * Significant group differences for Q1 (F(2,230) = 4.431 *p* = 0.013), Q5 (F(2,230) = 9.731 *p*<0.001) and Q6 (F(2,230) = 4.599 p = 0.011).

An overview of the questions addressed while discussing PS exposure in children with parents is presented in Figure 2. Questions about in-house smoking, smoking in the presence of the child, and the presence of smokers in the family were most frequently asked (respectively 73%, 69%, and 62% of the physicians always asked these questions). Next to these questions, physicians asked about parents' awareness on the health consequences (asked always by 40% of the physicians) and whether they took measures to prevent PS exposure in their children (asked always by 25% of the physicians). Merely 19% of the physicians always asked about smoking in the family car.

With regard to informing parents about the health risks for children due to PS exposure; of all physicians, 48% always, 45% often, 7% occasionally, and less than 1% never explained the risks of PS exposure for the child. Most often respiratory problems like a higher risk of asthma (80%), and the association between PS exposure and infections such as pneumonia, bronchitis, and bronchiolitis (81%) were mentioned by the physicians. An increased risk of sudden infant dead syndrome (SIDS), more frequent presentation of otitis media with effusion (OME), an increased risk of a loss in lung function, long-term risks like cancer and heart diseases, and an increased risk of active smoking of the child at a later age, were respectively mentioned by 20%, 33%, 52%, 39%, and 42% of all physicians. Furthermore, advising parents to stop smoking in the presence of their child was done by 53% of the physicians always and 29% of the physicians often.

There were some differences between the three health professions. Paediatricians questioned parents more frequently about other smokers in the house compared to the FPs (*p* = 0.011). Compared to the FPs, YHCPs reported to question parents more frequently about preventive strategies to protect children from PS exposure (*p*<0.001), and parental awareness about the health consequences of PS in children (*p* = 0.010).

### Facilitators

**Figure 3:**
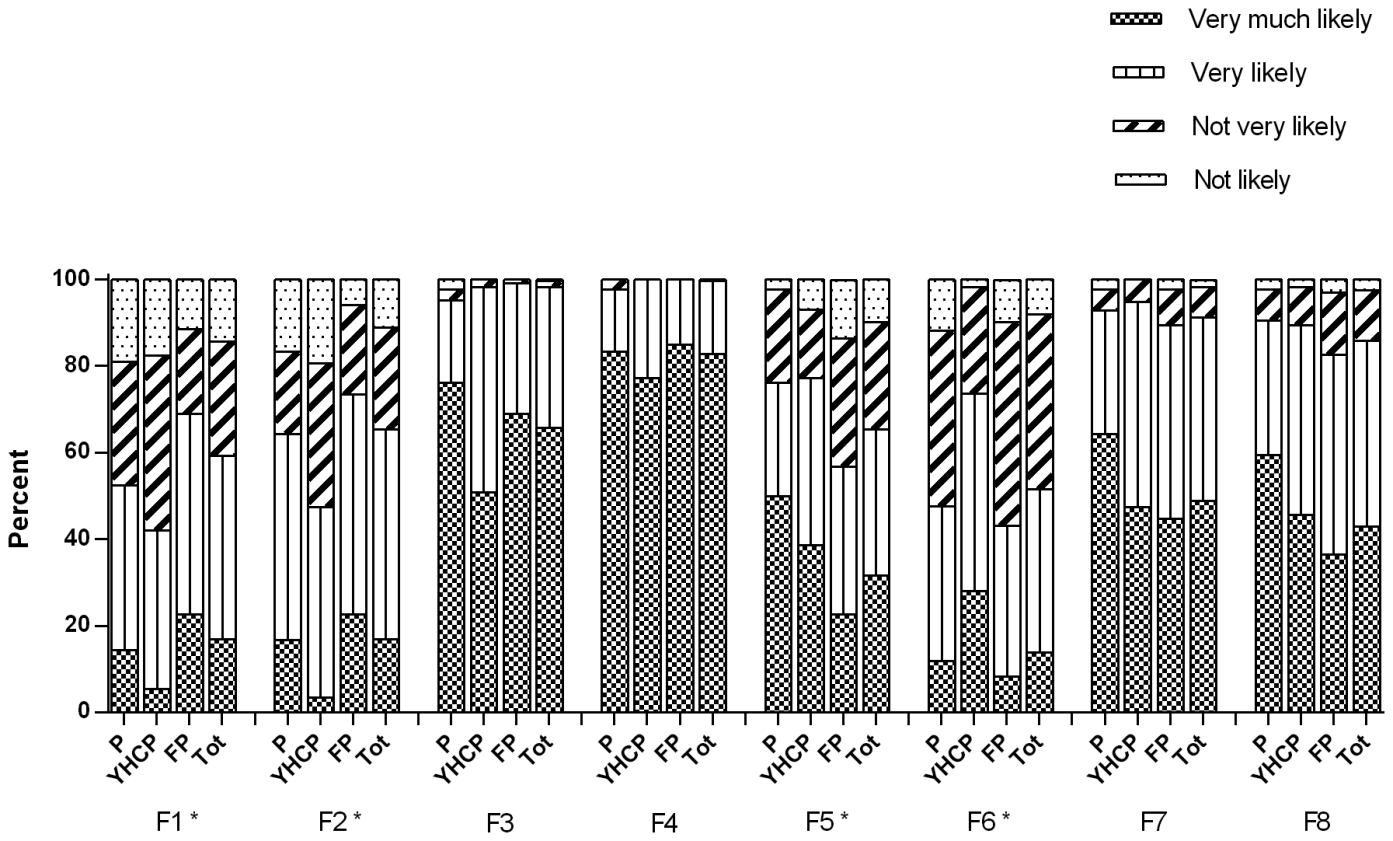
Facilitators. F1) A patient that you know for a longer time. F2) A patient that you see with a higher frequency of visits. F3) A child with known increased risk of respiratory diseases. F4) A child that presents for consultation with asthmatic complaints. F5) A family with a history of sudden infant death syndrome. F6) A child that presents for consultation with otitis media with effusion. F7) The smell of tobacco around the child and/or parents. F8) Parents with visible presence of smoking accessories. (P  =  paediatricians; YHCPs  =  youth health care physicians; FPs  =  family physicians; Tot  =  total). *Significant group differences for F1 (F(2,230) = 6.812 *p* = 0.001), F2 (F(2,230) = 9.673 *p*<0.001), F5 (F(2,230) = 7.978 *p*<0.001) and F6 (F(2,230) = 11.050 *p*<0.001).

The frequencies for each facilitator are shown in Figure 3. Very (much) likely to facilitate discussing PS exposure in children with parents was a child presenting with asthmatic complaints or a child with known increased risk of respiratory illness in respectively 100%, and 98% of all physicians. The smell of tobacco around the child and/or parents, and parents with visible smoking accessories were also very (much) likely to facilitate counselling for PS exposure in children in respectively 91% and 86% of all physicians. Consultation with a parent who is known for a longer time, a parent that is seen with a higher frequency of visits for their child, a family with a history of SIDS, and a child that presents with OME, were very (much) likely facilitators for physicians to discuss PS exposure in respectively 59%, 65%, 65%, and 52% of all physicians.

Comparisons between the three health professions showed that a patient that is known for a longer time was more likely to facilitate discussing PS exposure with parents for FPs compared to YHCPs (*p* = 0.002). A family with a history of SIDS was more likely to facilitate discussing PS in children with parents for paediatricians compared to FPs (*p* = 0.002) and YHCPs (*p* = 0.014). YHCPs found it easier to discuss PS exposure in children when a child presented with complaints of OME than paediatricians (*p* = 0.004) and FPs (*p*<0.001).

In the total group of physicians, the odds of providing advice were significantly increased when a child presented with otitis media (ORa 3.82; 95% CI 2.09–7.00); smell of tobacco around the child and/or parents (ORa 15.43; 95% CI 2.00–119.12); and in the case of parents with visible smoking accessories (OR a 6.94; 95% CI 2.32–20.78) (see Table 2).

**Table 2:** Relationship between each facilitator and providing advice.

	Unadjusted OR (95% CI)	Adjusted OR (95% CI)
***A patient that is known for a long time:***		
Not (very) likely	reference	reference
Very (much) likely	1.23 (0.72–2.08)	1.25 (0.71–2.20)
***A patient with high frequency of visits:***		
Not (very) likely	reference	reference
Very (much) likely	1.19 (0.69–2.05)	1.09 (0.61–1.95)
***A patient with known increased risk of respiratory diseases:***		
Not (very) likely	reference	reference
Very (much) likely	0.78 (0.11–5.66)	0.40 (0.03–5.29)
***A child with increased risk of asthma:***		
Not (very) likely	reference	reference
Very (much) likely	0.79 (0.05–12.70)	-
***A family with previous history of sudden infant dead syndrome:***		
Not (very) likely	reference	reference
Very (much) likely	1.60 (0.92–2.78)	1.80 (0.99–3.23)
***A child with otitis media with effusion:***		
Not (very) likely	reference	reference
Very (much) likely	3.05 (1.78–5.22)*	3.82 (2.09–7.00)*
***The smell of tobacco around child and/or parents:***		
Not (very) likely	reference	reference
Very (much) likely	17.30 (2.28–131.58)*	15.43 (2.00–119.12)*
***Visible presence of smoking accessories:***		
Not (very) likely	reference	reference
Very (much) likely	7.04 (2.39–20.75)*	6.94 (2.32–20.78)*

OR  =  odds ratio; 95% CI  =  95% confidence interval; * p<0.05; Adjusted for: sex, specialism, education on PS counselling, current smoking; “-” logistic regression analysis not possible due to small sample size.

### Barriers

**Figure 4:**
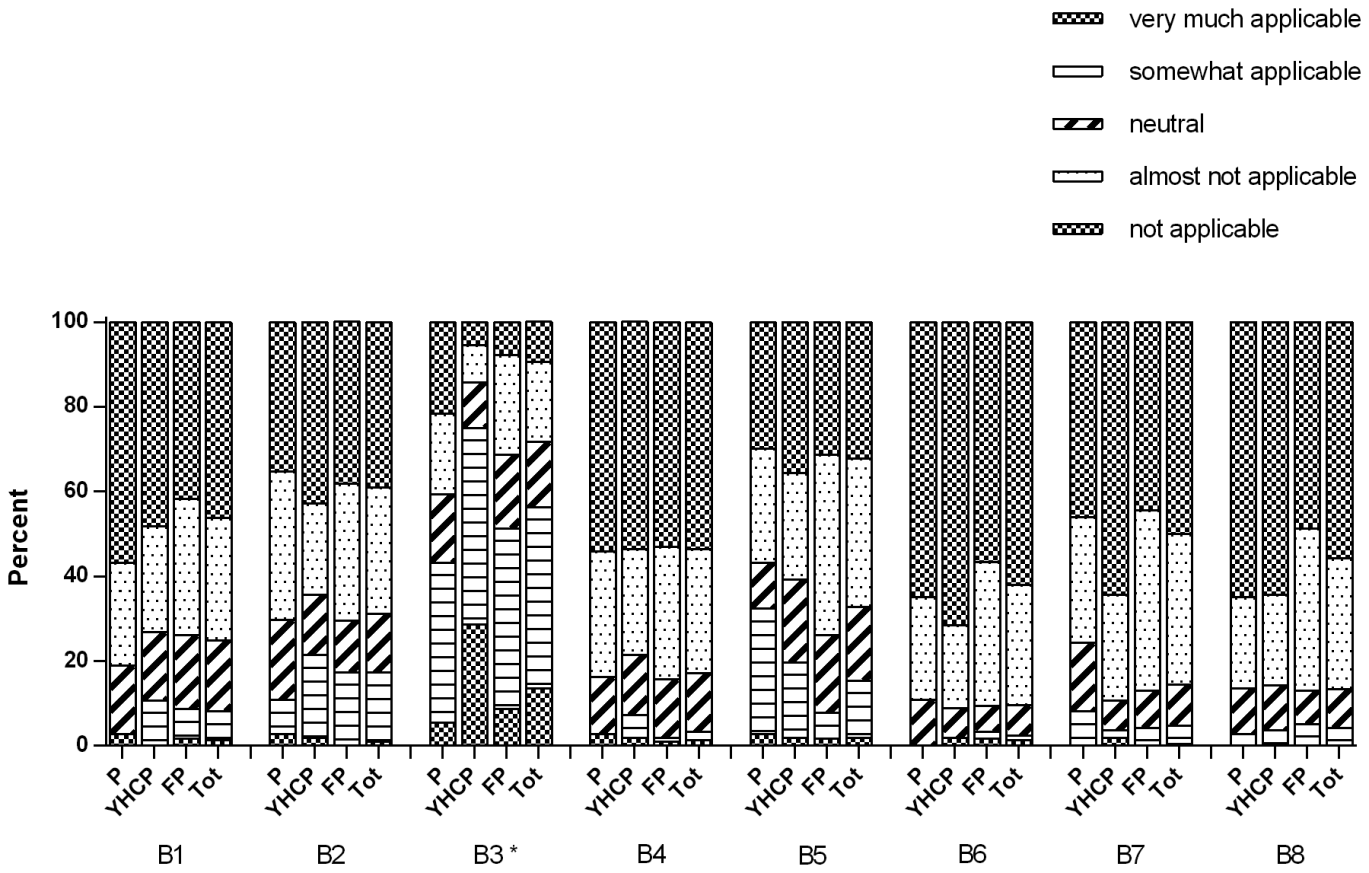
Barriers. B1) By talking about this topic I am invading the parents' privacy. B2) I expect that talking about this topic will damage the doctor-patient relationship. B3) I have none/little time to bring up this topic during consultation. B4) I do not find this topic important enough to discuss during consultation. B5) It has no effect to address this topic during consultation as there will be no change for the child anyways. B6) I do not see it as my responsibility to talk about this topic during consultation. B7) I do not have enough knowledge about this topic to bring it up during consultation. B8) I do not have enough communications skills to address this topic during consultation. (P  =  paediatricians; YHCPs  =  youth health care physicians; FPs  =  family physicians; Tot  =  total). *Significant group differences for B3 (F(2,207) = 8.551 *p*<0.001).

The frequencies for each barrier are shown in Figure 4. Lack of time was the most mentioned barrier and was reported as very much or a somewhat applicable in 56% of all the physicians. Furthermore, 17% of all the physicians reported fear of damaging the doctor-patient relationship as very much or somewhat applicable barrier. Low expectations regarding the efficacy of parental counselling for PS exposure in children was a very much or somewhat applicable barrier in 15% of the physicians. All other barriers included in this study reported to be less or not applicable, with frequencies below the 5%.

Only lack of time revealed significant differences between the health professions. This was significantly more frequent reported as very (much) applicable by YHCPs compared to paediatricians (*p*<0.001) and FPs (*p* = 0.005). An overall evaluation of the barriers and their association with addressing PS exposure in children showed that, lack of time was negatively associated with addressing PS exposure in children, but only in the unadjusted logistic regression analyses (OR 0.51; 95% CI 0.12–0.94) (see Table 3). The odds of addressing PS exposure in children were decreased if the physicians did not find the topic important enough (ORa 0.36; 95% CI 0.13–1.00) or if they did not belief in effectiveness of addressing PS in children (OR 0.41; 95% CI 0.17–0.99).

**Table 3:** Relationship between barrier and providing advice.

	Unadjusted OR (95% CI)	Adjusted OR (95% CI)
**Invading parents privacy:**		
Not (very) applicable	reference	reference
Neutral	0.54 (0.24–1.24)	0.60 (0.26–1.39)
Very (much) applicable	0.50 (0.16–1.61)	0.50 (0.15–1.66)
**Fear of damaging the doctor-patient relationship:**		
Not (very) applicable	reference	reference
Neutral	0.59 (0.24–1.48)	0.54 (0.21–1.37)
Very (much) applicable	1.33 (0.63–2.80)	1.31 (0.61–2.83)
**Lack of time:**		
Not (very) applicable	reference	reference
Neutral	0.76 (0.32–1.81)	0.85 (0.35–2.11)
Very (much) applicable	0.51 (0.27–0.97)*	0.51 (0.26–1.01)
**Topic not important enough during consultations:**		
Not (very) applicable	reference	reference
Neutral	0.34 (0.12–0.94)*	0.36 (0.13–1.00)*
Very (much) applicable	0.55 (0.11–2.78)	0.72 (0.12–4.15)
**No belief in effectiveness of addressing PS exposure:**		
Not (very) applicable	reference	reference
Neutral	0.41 (0.17–0.99)*	0.42 (0.17–1.04)
Very (much) applicable	0.85 (0.38–1.89)	0.74 (0.32–1.75)
**Do not see it as their responsibility:**		
Not (very) applicable	reference	reference
Neutral	0.50 (0.13–1.84)	0.47 (0.12–1.83)
Very (much) applicable	1.22 (0.20–7.45)	1.29 (0.21–8.05)
**Lack of knowledge for addressing PS exposure in children:**		
Not (very) applicable	reference	reference
Neutral	0.61 (0.21–1.78)	0.62 (0.20–1.92)
Very (much) applicable	0.19 (0.02–1.54)	0.17 (0.02–1.44)
**Lack of communication skills for addressing PS exposure in children:**		
Not (very) applicable	reference	reference
Neutral	0.65 (0.22–1.90)	0.76 (0.25–2.30)
Very (much) applicable	-	-

OR  =  odds ratio; 95% CI  =  95% confidence interval; PS =  passive smoke.

* p<0.05; Adjusted for: sex, specialism, education on PS exposure counselling, current smoking; “-” logistic regression analysis not possible due to small sample size.

### Amount of facilitators and barriers

Table 4 and Table 5 provide the frequencies of reported facilitators and barriers respectively for the physicians. Thirty percent of the physicians reported to have no barriers for addressing PS exposure in children. About 58% of the physicians reported to have at least one facilitator and at least one barrier for parental counselling for PS exposure in children. The combined number of facilitators and barriers for the remaining group of 3% is unknown due to missing values. The association between the amount of facilitators and addressing PS exposure was only significant in the group of physicians who had all 8 facilitators. There were no association between the amount of barriers and addressing PS exposure smoking.

**Table 4:** Number of reported facilitators and their association with addressing passive smoke (PS) exposure.

Amount of facilitators	Addressing PS exposure (n (%))			Association (OR (95%))
	Yes	No	Total	
0	0	0	0	-
1	0	0	0	-
2	0	1 (0)	1 (0)	-
3	0	9 (4)	9 (4)	-
4	5 (2)	12 (5)	17 (7)	Reference
5	15 (6)	23 (10)	38 (16)	1.56 (0.46–5.35)
6	28 (12)	47 (20)	75 (32)	1.43 (0.46–4.49)
7	24 (10)	24 (10)	48 (21)	2.40 (0.73–7.86)
8	31 (13)	15 (6)	46 (20)	4.96 (1.48–16.66)*
Total#	103 (44)	131 (56)	234 (100)	

# Numbers do not add up to 245 due to the exclusion of 11 physicians who never address PS exposure in children. OR  =  Odds Ratios; 95% CI  =  95% confidence interval; *p<0.05.

**Table 5:** Number of reported barriers and their association with addressing passive smoke (PS) exposure.

Amount of barriers	Addressing PS exposure (n (%))			Association (OR (95%))
	Yes	No	Total	
0	26 (12)	37 (18)	63 (30)	Reference
1	30 (140	54 (26)	84 (40)	0.79 (0.40–1.55)
2	13 (6)	32 (15)	45 (21)	0.58 (0.26–1.31)
3	3 (1)	12 (6)	15 (7)	0.36 (0.09–1.39)
4	1 (0)	2 (1)	3 (1)	0.71 (0.06–8.27)
5	0	1 (0)	1 (0)	-
6	0	0	0	-
7	0	0	0	-
8	0	0	0	-
Total#	73 (35)	138 (65)	211 (100)	

# Numbers do not add up to 245 due to the exclusion of 27 physicians who always address PS exposure in children, and 7 physicians who did not complete the survey. OR  =  Odds Ratios; 95% CI  =  95% confidence interval.

### Education about PS

Twenty four per cent of the responders had received postgraduate education about PS exposure. From this subgroup, 13% always, 38% often, 46% occasionally, and 4% never discussed PS exposure in children during a consultation. These answers did not differ significantly from physicians who did not have any education about PS exposure. Of those without PS exposure related education, 49% were interested in receiving more education. The highest percentages among all physicians who would like to get education about PS exposure were found in the groups discussing PS exposure occasionally (48%) or never (55%).

### Non-responders

The response rate of the abbreviated version of the questionnaire was 38% (n = 54 of the 144 physicians that received an invite to complete the abbreviated questionnaire). The responders were 15% paediatricians, 7% YHCPs and 78% FPs. Their main reason for not participating with the initial electronic questionnaire was lack of time. Parental counselling for PS exposure in children was reported in 1.9% of the physicians always, 30% often, 67% occasionally, and 2% never. The frequency for addressing PS exposure was not statistically different between the two groups of responders (*p* = 0.055). Compared to the responders group, considerably fewer physicians in the non-responding group (28%) would like more education about PS exposure counselling.

## Discussion

Our findings showed that most physicians addressed PS exposure in children; 11% always, and 85% addressed PS exposure often to occasionally. There were no differences between the three health professions in this regard. In 1994, 62% of Dutch child health professionals reported that prevention of PS exposure was not included in their policy [14]. Therefore, this study suggests that physicians engage in parental counselling for PS exposure in children more frequently than several years ago.

In this study, parental awareness about the health effects of PS exposure in children was more regularly addressed than strategies to prevent it. The YHCPs questioned parents more frequently about preventive strategies to protect children from PS exposure and about their knowledge concerning the health consequences of PS exposure in children. Possibly because prevention is one of the key tasks of the YHCPs and they reported to have more education on PS exposure counselling.

Generally, the most important facilitators regarding parental counselling for PS exposure in children were children with asthmatic complaints or increased risk of respiratory diseases. This was also observed in other studies [11], [25], [27]. However, waiting until a child has a respiratory disease to educate parents might be unethical. Furthermore, the FPs reported to find it easier to counsel a family, for PS exposure in children that is known for a longer time. Compared to the other professions, FPs may see families more frequently which gives them an opportunity to build a closer relationship and therefore facilitate PS counselling.

Similarly to other studies, [10], [11], [12], [13], [14] the most mentioned barrier for physicians to discuss PS exposure in children was lack of time. It might be understandable, but studies suggest that even a brief advice about PS exposure in children can be an effective first step to motivate parents to change their smoking behaviour [21], [28].

Looking from a behavioural perspective [29], the study may suggest that besides the limiting factor of lack of time, physicians might be dealing with other factors influencing their decision to counsel parents for PS exposure in children. Most physicians felt responsible for addressing PS exposure in children. A smaller percentage of paediatricians in the study reported to find it their responsibility to address PS exposure in children compared to the other professions. This observation could be explained by the small sample of paediatricians who participated in the study, when compared to the other two professions. Nevertheless, not finding it their responsibility was not a barrier for them to address PS exposure. Interestingly, only 5% of all the physicians thought it was the responsibility of the paediatricians to address PS exposure in children. Probably because in the Netherlands children are seen by paediatricians after referral from FP, and the paediatricians will therefore focus on solving the health problem for which referral was necessary.

In the last years media attention on PS exposure has increased, including worldwide measures to prevent PS exposure in public places. Moreover, parents indicated that they expect physicians to counsel them on behavioural changes that could improve the health of their children [9]. This may reassure physicians to include PS exposure counselling during consultations. However, though they expect counselling for PS exposure in children, they might still not embrace such counselling.

Currently, the prevalence of PS exposure in children is particularly high in social and economically deprived families [6], [30], [31]. Counselling such families might be more challenging. In a study by Browning et al, health care providers were less likely to assist in smoking cessation in disadvantaged socioeconomic groups [32]. Moreover, Binns et al suggested that a brief communication for PS exposure in children with parents of low socioeconomic status might not be effective [33].

Contrary to previous studies, the physicians in our study reported to have proper knowledge and communication skills to address PS exposure [13], [34]. YHCPs were more likely to have participated in postgraduate education about PS exposure. We expected this group to be more confident and therefore discuss PS exposure more often, but this was not supported by our data. Probably they do not address PS exposure more frequently due to lack of time, which they reported more often as a barrier than the other professions. Furthermore, the YHC nurses usually see the children before the YHCPs, and they address most educational issues with parents while the YHCPs focus on more complicated health care issues. In the Netherlands, nurses working in well-baby clinics provided more PS exposure counselling to parents when compared to the YHC physicians [35]. Also, YHCPs use questionnaires, partly as a screening tool, which are completed by parents prior to a consultation. Therefore, perhaps the YHCPs only address PS exposure in children when parents answered to smoke at home and/or when children have respiratory problems. Compared to the YHCPs of our study, physicians of a Canadian study with postgraduate tobacco-related education were more likely to address PS exposure in children with and without respiratory symptoms when compared to physicians without extra education [11]. But in the current study no difference were seen between the physicians who received post-graduate education about PS exposure and those who did not.

Remarkably, about half of the physicians who did not discuss PS exposure always would like to receive more education on this subject. Their exact reasons are unknown; our questionnaire did not inquire this. Contrary, lack of knowledge and communication skills were not barriers for addressing PS exposure. So, why do they want more education? Possibly they are overestimating their own communication skills when filling out barrier items [15], or may be limited due to beliefs of low-self efficacy towards parental counselling for PS exposure in children. Several studies showed that paediatricians and FPs significantly increased their self-efficacy for parental counselling for PS exposure after a brief training about the effects of PS exposure and how to address PS exposure to parents [36], [37]. Furthermore, physicians with continued postgraduate tobacco related education were more likely to counsel parents compared to physicians who did not receive additional training after medical school [11].

### Limitations

The sample size of 245 was modest, including the response rate of 34% when compared to other studies [10], [11], [14], [25]. There were no differences in the response rates and the frequencies of addressing PS exposure in children between the physicians who completed the initial questionnaire and those who completed the short questionnaire (non-responders), therefore selection bias was unlikely. Bias due to social desirable answers could have occurred, which emphasizes that the physicians may be counselling parents for PS exposure in children less frequently than what has been reported in this study. Still, participation was voluntary and the questionnaires were processed anonymously.

### Recommendations

More in depth data collection among physicians working in child-care settings could provide more insights about the factors associated with parental counselling for PS exposure in children. Furthermore, a validated and reliable instrument to measure physicians' practices regarding parental counselling for PS exposure in children should be developed to enable the generalizability of similar studies and to better measure change over time. To compensate for the lack of time physicians often have to deal with (although this is probably the case already) more intensive counselling of PS exposure may also be given through trained nurses. Future studies should also evaluate the practices of the Dutch nurses in providing PS exposure counselling to parents. We also recommend investing more in education programs about PS exposure in children for relevant professionals.

## Conclusion

This study showed that Dutch physicians working in the child-care settings could counsel parents for PS exposure in children more frequently. Only 11% of the physicians who participated in this study reported to discuss PS exposure in children always. This is a point of concern, since Dutch smokers are more ignorant about the harmful effects of PS exposure to others. There were no differences between the three health professions regarding their frequently of addressing PS exposure in children. Lack of time appeared to be the most often mentioned barrier and physicians were more likely to counsel parents for PS exposure in children with respiratory complaints. Additionally, a need for more postgraduate education on parental counselling for PS exposure was expressed.

## Supporting Information

Questionnaire S1 **Supplement questionnaire.**
(DOC)
