# Supplementary material for: Addressing Passive Smoking in Children
Source: PLoS One. 2014 May 8;9(5):e93220. doi: 10.1371/journal.pone.0093220 (PMC4014468; doi:10.1371/journal.pone.0093220)
Supplement: Questionnaire S1 — Supplement questionnaire. (DOC) [file pone.0093220.s001.doc]

**Supplement:** **Sample of the most important questions:**

What is your gender?

| - Female | - Male |
| --- | --- |

What is your specialization?

| - Paediatrics | - Child and youth health care | - General practice | - Other |
| --- | --- | --- | --- |

**For how long are you working in your specialization?**

| - Less than 1 year - 1-2 years - 3-5 years - 6-10 years - 11-15 years - More than 15 years |
| --- |

Do you discuss passive smoke exposure in children during a consult?

| - Always | - Often | - Occasionally | - Never |
| --- | --- | --- | --- |

**Facilitators**

**If you address passive smoke exposure in children during a consultation, could you indicate to what extent the following situations will make it more likely for you to bring up the topic during consultation?**

|  | *Very much likely* | *Very likely* | *Not very likely* | *Not likely* |
| --- | --- | --- | --- | --- |
| A patient that you know for a longer time |  |  |  |  |
| A patient that you see with a higher frequency of visits |  |  |  |  |
| A child with known increased risk of respiratory diseases |  |  |  |  |
| A child that presents for consultation with asthmatic complaints |  |  |  |  |
| A family with a history of sudden infant death syndrome(SIDS) |  |  |  |  |
| A child that presents for consultation with otitis media with effusion (OME) |  |  |  |  |
| The smell of tobacco around the child and/or parents |  |  |  |  |
| Parents with visible presence of smoking accessories |  |  |  |  |

**Topics discussed** *(for all physicians expect those who answered never to give counselling for passive smoke exposure in children)*

Could you indicate which questions you ask your patients?

|  | *Always* | *Often* | *Occasionally* | *Never* |
| --- | --- | --- | --- | --- |
| Does any member of your family smoke? |  |  |  |  |
| Does anyone smoke inside the house? |  |  |  |  |
| Does anyone smoke in the presence of your child? |  |  |  |  |
| Does anyone smoke inside your family car? |  |  |  |  |
| Did you take any efforts to prevent passive smoking of your child? |  |  |  |  |
| Are parent(s)/carer(s) aware of the health consequences of passive smoking for the child? |  |  |  |  |

Do you give information about the health consequences for children due to passive smoke exposure?

| - Always | - Often | - Occasionally | - Never |
| --- | --- | --- | --- |

*Could you indicate which health consequences you address?* (all physicians except those who answered ‘never’ to the previous question)

| - Increased risk of sudden infant dead syndrome - More frequent occurrences of otitis media with effusion - Strong relationship between passive smoke exposure and respiratory infections like pneumonia, bronchitis and bronchiolitis - Increased risk of asthma - Increased risk of a decreased lung function - Long term risks of passive smoking like lung cancer and heart diseases - Higher probability a child starts smoking - Other, namely … |
| --- |

If you address passive smoke exposure in children during a consultation, do you advise smokers in the family to stop smoking in the presence of the child?

| - Yes | - No |
| --- | --- |

**Barriers**

**In case you do not address passive smoke exposure in children during a consultation, can you indicate which of the following considerations may play a role?**

|  | *Very much applicable* | *Somewhat applicable* | *Neutral* | *Not very applicable* | *Not applicable* |
| --- | --- | --- | --- | --- | --- |
| By talking about this topic I am invading the parents privacy |  |  |  |  |  |
| I expect discussing this subject will damage the doctor-patient relationship |  |  |  |  |  |
| I have none/little time to bring up this topic during consultation |  |  |  |  |  |
| I do not find this topic important enough to discuss during consultation |  |  |  |  |  |
| It has no effect to address this topic during consultation as there will be no change for the child anyways |  |  |  |  |  |
| I do not see it as my responsibility to talk about this topic during consultation |  |  |  |  |  |
| I do not have enough knowledge about this topic to address it during consultation |  |  |  |  |  |
| I have too little communications skills to address this topic during consultation |  |  |  |  |  |

Could you indicate to which degree you have difficulties to address the following subjects in a consult?

|  | *Very easy* | *Easy* | *Neutral* | *Difficult* | *Very difficult* |
| --- | --- | --- | --- | --- | --- |
| Overweight and/or obesity in children |  |  |  |  |  |
| Neglect of children by parents |  |  |  |  |  |
| Child abuse |  |  |  |  |  |
| Incest |  |  |  |  |  |
| Passive smoke exposure in children |  |  |  |  |  |
| Addiction(s) from parent(s)/carer(s) |  |  |  |  |  |

From your point of view, who is responsible for discussing passive smoke exposure in children?

| - Paediatrician - Doctor of child and youth health care - General practitioner - Other, namely … |
| --- |

Do you smoke?

| - Yes | - No |
| --- | --- |

Did you suffer from passive smoke exposure during your childhood (0-18 years)?

| - Yes | - No |
| --- | --- |

Did you ever receive postgraduate education about passive smoke exposure in children?

| - Yes | - No |
| --- | --- |

Would you be interested in getting (more) postgraduate education about passive smoke exposure in children?

| - Yes | - No |
| --- | --- |

1. Barnes Dodge RA, Cabana MD, O'Riordan MA, Heneghan A (2008) What factors are important for pediatric residents' smoking cessation counseling of parents? Clin Pediatr (Phila) 47: 237-243.

2. Pérez-Stable EJ, Juarez-Reyes M, Kaplan C, Fuentes-Afflick E, Gildengorin V, et al. (2001) Counseling smoking parents of young children: comparison of pediatricians and family physicians. Archives of pediatrics & adolescent medicine 155: 25-31.

3. Victor JC, Brewster JM, Ferrence R, Ashley MJ, Cohen JE, et al. (2010) Tobacco-related medical education and physician interventions with parents who smoke: Survey of Canadian family physicians and pediatricians. Can Fam Physician 56: 157-163.

4. Collins BN, Levin KP, Bryant-Stephens T (2007) Pediatricians' Practices and Attitudes about Environmental Tobacco SMoke and Parental Smoking. The Journal of Pediatrics: 547-552.
